# Increased Prevalence of Liver Fibrosis and HIV Viremia among Patients with HIV, HBV, and Tuberculosis in Botswana

**DOI:** 10.3390/pathogens9110950

**Published:** 2020-11-14

**Authors:** Bonolo B. Phinius, Motswedi Anderson, Lynnette Bhebhe, Kabo Baruti, Godiraone Manowe, Wonderful T. Choga, Lucy Mupfumi, Tshepiso Mbangiwa, Mbatshi Mudanga, Sikhulile Moyo, Richard Marlink, Jason T. Blackard, Simani Gaseitsiwe

**Affiliations:** 1 Research Laboratory, Botswana Harvard AIDS Institute Partnership, Gaborone 0000, Botswana; bphinius@bhp.org.bw (B.B.P.); manderson@bhp.org.bw (M.A.); lbhebhe@bhp.org.bw (L.B.); kabobaruti@gmail.com (K.B.); gmanowe@bhp.org.bw (G.M.); wchoga@bhp.org.bw (W.T.C.); lmupfumi@bhp.org.bw (L.M.); tmbangiwa@bhp.org.bw (T.M.); mmudanga@bhp.org.bw (M.M.); smoyo@bhp.org.bw (S.M.); 2 Department of Biological Sciences, Faculty of Sciences, University of Botswana, Gaborone 0000, Botswana; 3 Department of Pathology, Faculty of Health Sciences, University of Cape Town, Cape Town 7925, South Africa; 4 Department of Statistics, Faculty of Sciences, University of Botswana, Gaborone 0000, Botswana; 5 Department of Immunology and Infectious Diseases, Harvard T.H. Chan School of Public Health, Boston, MA 02115, USA; 6 Rutgers Global Health Institute, Robert Wood Johnson Medical School, Rutgers University, New Brunswick, NJ 08901, USA; rmarlink@globalhealth.rutgers.edu; 7 Division of Digestive Diseases, University of Cincinnati College of Medicine, Cincinnati, OH 45267, USA; jason.blackard@uc.edu

**Keywords:** hepatitis B virus, tuberculosis, human immunodeficiency virus, Botswana, Africa

## Abstract

People with concomitant human immunodeficiency virus (HIV) and tuberculosis (TB) have an increased risk of hepatotoxic reactions due to antiretroviral therapy (ART) and anti-TB therapy (ATT). Concomitant hepatitis B virus (HBV) in these patients may lead to poorer health outcomes. To assess liver enzyme levels and immune response in adults with HIV, HBV, and TB, data from 300 antiretroviral-naïve people living with HIV (PLWHIV) were analyzed. The prevalence of HIV/HBV (cHIV/HBV) and HIV/TB (cHIV/TB) was 28% (95% CI: 23.0–33.4) and 10% (95% CI: 6.8–14.0), respectively. HIV/HBV/TB (cHIV/HBV/TB) prevalence was 5.3% (95% CI: 3.1–8.5). There was a statistically significant difference between the groups of participants in HIV viral load (*p* = 0.004), hemoglobin levels (*p* = 0.025), and body mass index (*p* = 0.011). A larger proportion of cHIV/HBV/TB participants (37.5%) had an aspartate aminotransferase to platelet ratio index (APRI) score ≥0.5 (*p* = 0.013), a lower cutoff for significant liver fibrosis. Immunological non-responders (CD4+ T-cell count <20% gain and HIV viral load <400 copies/mL at 6 months) were observed in all groups except those with cHIV/TB. Our findings support the need to screen for infections that could cause excessive liver damage prior to ATT or ART initiation, such as HBV.

## 1. Introduction

Globally, approximately 251,000 tuberculosis (TB)-associated deaths were recorded among people living with human immune deficiency virus (PLWHIV) in 2018 with a high proportion of people with concomitant HIV/TB (cHIV/TB) occurring in Africa [1]. Botswana is no exception and is listed among the thirty highest TB/HIV burdened countries by the World Health Organization (WHO) [1]. Other HIV co-infections such as viral hepatitis, particularly hepatitis B virus (HBV) is highly prevalent in Africa with approximately 71% of people with concomitant HIV/HBV (cHIV/HBV) reported in sub-Saharan Africa [2]. Botswana also has one of the highest reported HBV incidences among PLWHIV in Southern Africa [3], and prevalence rates vary from 3.1% to 10.6% in different at-risk populations [4,5,6,7,8,9]. Focusing efforts on preventive measures such as vaccinations and early diagnosis is of great importance. 

Although treatment of HIV co-infections has been shown to decrease HIV viral load [10], other multiple undiagnosed infections could counteract this effort and possibly lead to delayed immune recovery. Currently, there are limited data on the burden of multiple infections with TB and HBV among PLWHIV in a high HIV endemic country such as Botswana. HIV, HBV, and TB multi-infection burden is rarely studied in sub-Saharan Africa; however, a prevalence of 7.5% was reported in China [11] indicating a possibly overlooked source of adverse health outcomes in the context of HIV endemicity. 

HIV co-infections result in poor health outcomes that are not only a function of their pathogenesis but also the complications arising from their treatment. Patients with cHIV/TB are known to have increased hepatotoxic reactions particularly with the initiation of anti-TB therapy (ATT) [12,13]. Immune reconstitution inflammatory syndrome (IRIS) is also prevalent in patients with cHIV/TB at antiretroviral therapy (ART) initiation [14]. Chronic and occult HBV among PLWHIV may result in adverse health outcomes [15], and HIV treatments such as Truvada-based ART are efficacious in treating HBV [16]. The immense pressure on the liver resulting from disease and treatment therefore requires routine assessment for patients on ART and ATT. To assess liver damage, inexpensive non-invasive methods such as evaluating aspartate aminotransferase (AST), alanine aminotransferase (ALT), AST to platelet ratio index (APRI), and fibrosis-4 (FIB-4) are employed. These liver enzymes, in particular aminotransferases, are released in circulation after liver injury [17]. These methods are suitable in low- and middle-income countries with limited resources or expertise for other methods such as liver biopsy. 

Immune restoration in PLWHIV that could also be co-infected with other diseases is measured mainly by the use of CD4 T+ cell counts in resource-limited settings. It is especially important to assess immune response in these patients as liver cirrhosis has been associated with low CD4 T+ cell count even in the absence of HIV [18], as well as among PLWHIV who are treatment naïve indicating the possibility of HIV-associated liver damage [19]. To assess such dynamics in people with concomitant HIV, HBV, and TB (cHIV/HBV/TB), we determined the prevalence of cHIV/HBV/TB in an adult population initiating a Truvada-based ART in Botswana. We also aimed to evaluate the liver enzyme levels and immune response among PLWHIV only, participants with concomitant HIV and HBV (cHIV/HBV) and cHIV/TB, as well as participants with cHIV/HBV/TB. 

## 2. Materials and Methods 

### 2.1. Study Participants

The study participants were PLWHIV initiating Truvada-based ART in Botswana from a longitudinal study “Bomolemo” conducted at the Botswana Harvard AIDS Institute Partnership (BHP) between 2008 and 2011. The Bomolemo study recruited 309 ART-naive PLWHIV and screened 300 for HIV, HBV, and TB as previously described [20]. Eligibility criteria for the Bomolemo study included a minimum age of 18 years, presence of an AIDS defining condition, or CD4+ T-cell count of less than 250 cells/mm^3^. Female participants found to be pregnant or who had received a single dose of nevirapine through the prevention of mother to child transmission program within 6 months preceding enrollment were excluded. Written informed consent was obtained from study participants. The study received ethical approval from the Botswana Ministry of Health Research Development Committee (PPME-13/18/1) and the Harvard T.H. Chan School of Public Health Institutional Review Board (16470–02). 

### 2.2. Laboratory Testing

All laboratory tests were conducted at the Botswana Harvard HIV reference laboratory (BHHRL) following manufacturer’s instructions. HIV and hepatitis B virus surface antigen (HBsAg) were diagnosed using enzyme-linked immunosorbent assays (ELISA). HIV ELISA included a double/parallel testing algorithm with Vironostika HIV Uniform II plus O (BioMérieux France, Marcy l’Etoile, France) and Murex HIV-1.2.O (Biotech, Dartford, U.K.) as per manufacturer’s instructions. HBsAg ELISA was also carried out using the Murex HBsAg kit (Biotech, Dartford, U.K.). HBV DNA was quantified by use of COBAS^®^ AmpliPrep/COBAS^®^ Taqman^®^, HBV Test v.2.0 (Roche diagnostics, Mannheim, Germany). Occult HBV infection (OBI) was defined as negative HBsAg with a detectable HBV viral load. TB was confirmed using either a positive sputum acid-fast bacillus or culture result or an abnormal chest radiology as previously described [20]. HIV viral load was measured using the COBAS^®^ AmpliPrep/COBAS^®^ AMPLICOR^®^ HIV-1 MONITOR Test, version 1.5 (Roche Molecular Systems, Branchburg, NJ). CD4+ T-cell counts were measured on the BD FACScalibur platform (BD Biosciences, San Jose, CA, USA). Hematology tests were conducted on the Sysmex XE-2100 (Sysmex, Kobe, Japan). The COBAS INTEGRA 400 plus (Roche Diagnostics, Indianapolis, IN, USA) was used for the measurement of chemistry panels.

### 2.3. Outcome Definitions

Immunological non-response (INR) was defined as <20% increase in CD4+ T-cell count from the baseline CD4+ T-cell count to 6 months of follow-up [21] with an HIV viral load of less than 400 copies/mL as per the limit of detection [20]. Four non-invasive methods were used to assess liver damage at baseline (prior to ART initiation) in these patients, including AST, ALT, APRI, and the FIB-4 index. The upper limit of normal (ULN) for ALT and AST were considered to be 42 and 41 U/L respectively, as per the BHHRL protocols. AST and ALT grades were defined as grade 1 (1.25–2.5 × ULN), grade 2 (2.6–5.0 × ULN), grade 3 (5.1–10.0 × ULN), or grade 4 (>10.0 × ULN) per the Division of AIDS adverse event grading table [22]. APRI and FIB-4 scores were calculated as previously reported [23,24], and low cutoff values were used for the staging of fibrosis. The low cutoff value for APRI was 0.5, while the FIB-4 low cutoff value was 1.45 for liver fibrosis staging. The low cutoff for cirrhosis using APRI was 1.0, while the high cutoff was 2.0 [25].

### 2.4. Statistical Analysis

Kruskal–Wallis test was used to compare the median CD4+ T-cell count, log HIV viral load, ALT, AST, APRI, and FIB-4 levels across the different groups. Dunn’s test with Bonferroni correction was used to adjust for multi-comparison between groups. Pearson’s chi-squared test (χ²) was used for comparisons between categorical data. All statistical analysis was performed in STATA version 15.1 (Stata Corporation, College Station, TX, USA), and *p*-values less than 0.05 were considered statistically significant.

## 3. Results

### 3.1. Prevalence Rates of Co-Infections with HIV

**Figure 1:**
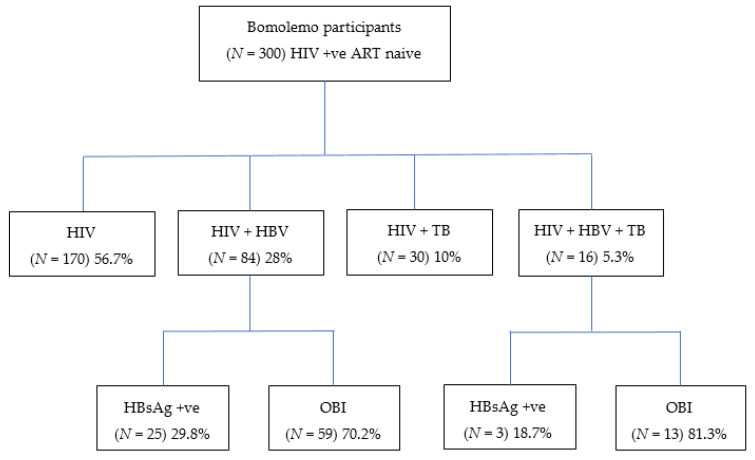
Proportion of Bomolemo participants with HIV, HBV, and/or TB co-infections. HIV; human immunodeficiency virus; HBV, hepatitis B virus; TB, tuberculosis; HBsAg, hepatitis B surface antigen; OBI, occult hepatitis B virus infection; +ve, positive.

Of the 300 participants at baseline, 56.7% [95% CI: 50.9–62.4] (170/300) were PLWHIV without HBV and TB. Twenty-eight percent [95% CI: 23.0–33.4] (84/300) of the participants had cHIV/HBV, 10% [95% CI: 6.8–14.0] (30/300) had cHIV/TB, and 5.3% [95% CI: 3.1–8.5] (16/300) were participants with cHIV/HBV/TB. Among those that had cHIV/HBV, 29.8% [95% CI: 20.3–40.7] (25/84) tested positive for HBsAg, while the remaining 70.2% [95% CI: 59.3–79.7] (59/84) had OBI (Figure 1). 

### 3.2. Participant Baseline Demographics and Clinical Characteristics

At baseline, there were no statistically significant differences across the study groups in median CD4+ T-cell count, ALT, AST, APRI, or FIB-4 levels. However, there was significant statistical difference in median hemoglobin levels (*p* = 0.025), median body mass index (BMI) (*p* = 0.011), and log HIV viral load (*p* = 0.004) at baseline (see Table 1). 

There was a statistically significant difference in the proportion of participants with previous exposure to HBV (HBcAb), (*p* = 0.001). There is evidence of previous exposure to HBV in PLWHIV and participants with cHIV/TB. However, the highest proportions were observed in participants with cHIV/HBV/TB (82.3%) and those with cHIV/HBV (69.1%), which is not surprising as some of the participants were also HBsAg positive. Five participants—4 with cHIV/HBV and 1 with cHIV/HBV/TB—were HBsAg positive but HBcAb negative (data not shown). There was no significant difference between participants with cHIV/HBV who had chronic infection versus OBI for all variables analyzed (data not shown). 

**Figure 2:**
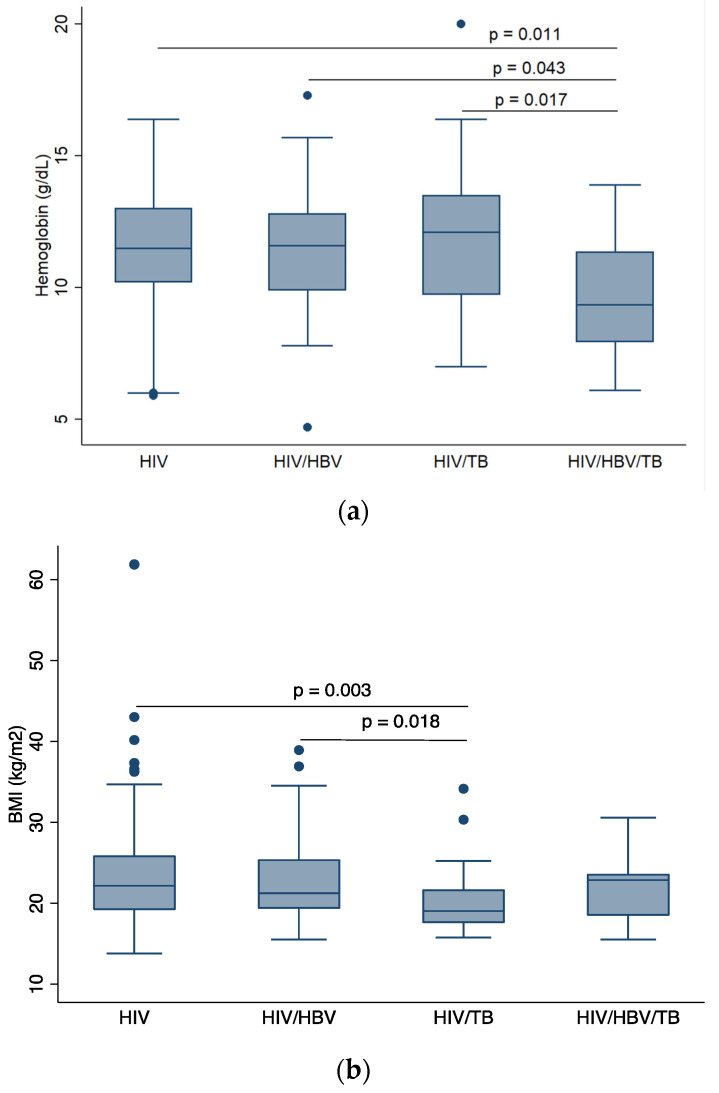

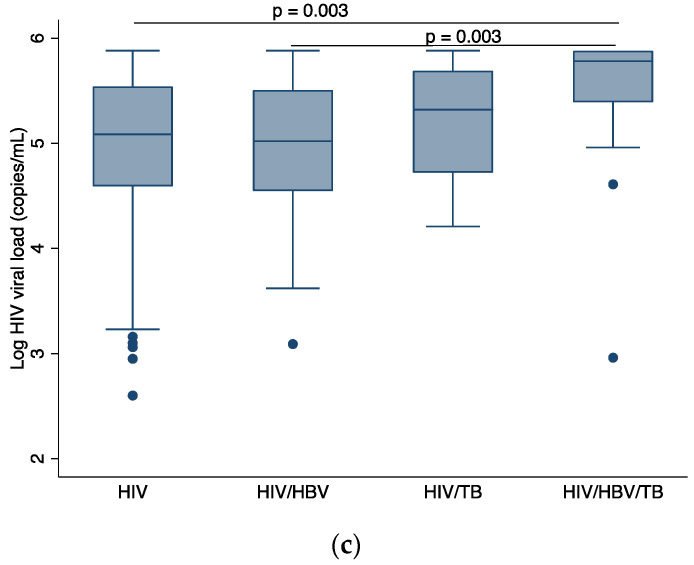
Comparison of baseline demographics across participant groups, (**a**) comparison of median BMI across participant groups, (**b**) comparison of median hemoglobin level across participant groups, (**c**) comparison of median log HIV viral load across participant groups. HIV: human immunodeficiency virus; HBV: hepatitis B virus; TB: tuberculosis; BMI: body mass index. *p*-values are by Dunn’s test with Bonferroni correction. Only *p*-values that indicate statistically significant differences in median values between patient groups are shown.

Statistically significant differences in hemoglobin levels were observed between PLWHIV versus cHIV/HBV/TB (*p* = 0.011), cHIV/HBV versus cHIV/HBV/TB (*p* = 0.043), and cHIV/TB versus cHIV/HBV/TB (*p* = 0.017) groups as shown in Figure 2a. Figure 2b shows that cHIV/TB participants had lower BMI compared to PLWHIV (*p* = 0.003) and cHIV/HBV participants (*p* = 0.018). Participants with cHIV/HBV/TB had the highest log viral load compared to PLWHIV only (*p* = 0.003) and cHIV/HBV (*p* = 0.003).

### 3.3. Hepatotoxicity

**Figure 3:**
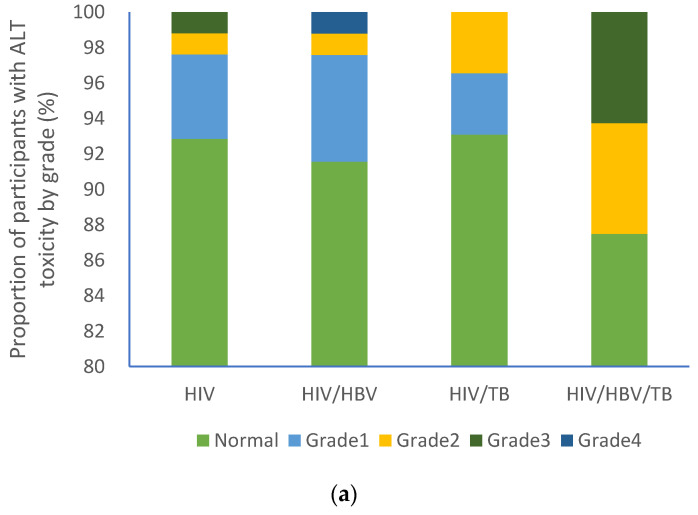

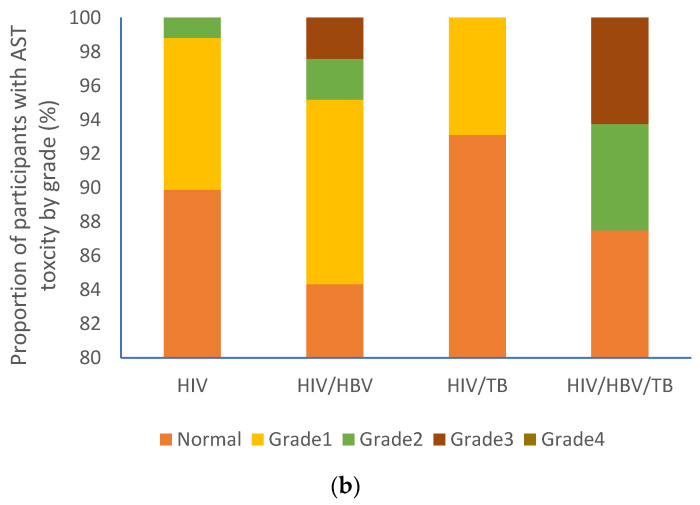
Proportion of participants with varying liver function test grades: (**a**) ALT grades and (**b**) AST grades. HIV: human immunodeficiency virus; HBV: hepatitis B virus; TB: tuberculosis; AST: aspartate aminotransferase; ALT: alanine aminotransferase.

At baseline, the majority of participants from each group had normal ALT (HIV (92.7%); HIV/HBV (91.8%); HIV/TB (93.1%); HIV/HBV/TB (87.5%) as shown in Figure 2a. A similar trend was observed with AST levels [HIV (89.9%%); HIV/HBV (84.3%); HIV/TB (93.1%); HIV/HBV/TB (87.5%)] as shown in Figure 2b. No participants had grade 4 AST toxicity, Figure 2b, whilst grade 4 ALT toxicity was recorded in one participant with cHIV/HBV, Figure 3a.

There was a significant statistical difference between the groups in APRI score categories (*p* = 0.013). A higher proportion of participants with cHIV/HBV/TB (37.5%) had an APRI score of greater than 0.5 compared to the other groups: HIV (16%), HIV/HBV (15.9%), and HIV/TB (10.7%) (Table 2).

These participants had APRI scores >1.0, which is a low cutoff for cirrhosis, a high cutoff being 2.0. The lowest APRI scores were among mono-infected HIV participants (BB1–BB3). Participants with cHIV/HBV had the highest APRI scores (BB8 and BB6). Participants BB6–BB10 had APRI scores of greater than 2.0 indicating significant liver cirrhosis. Participant BB6, deceased, had the highest APRI scores indicating severe liver damage (Table 3).

### 3.4. Immunological Response

**Figure 4:**
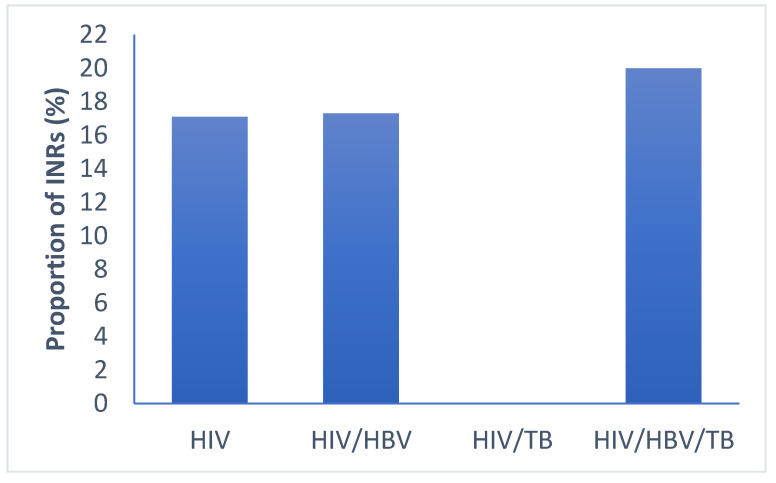
Proportion of immune-non-responders in the different groups of infections. HIV: human immunodeficiency virus; HBV: hepatitis B virus; TB: tuberculosis; INR: immunological non responders. *p*-values < 0.05 are statistical different by Pearson’s chi-squared test for categorical data.

All patient groups had low proportions of INR at 6 months of follow-up [HIV (17.1%, 28/164), HIV/HBV (17.3%, 14/81), HIV/TB (0% 0/29), HIV/HBV/TB (20%, 3/15)]. These proportions were not statistically different (*p* = 0.110) as shown in Figure 4.

## 4. Discussion

In this study conducted in a cohort of PLWHIV initiating ART in Botswana, we found a cHIV/HBV/TB prevalence of 5.3% with cHIV/HBV/TB participants having the lowest hemoglobin level and high HIV viral loads. Participants with cHIV/TB had a low BMI. Liver damage was more prevalent among participants with cHIV/HBV/TB at a low cutoff for fibrosis. In addition, immunological non-response was observed in all groups of participants at 17–20% apart from those with cHIV/TB. 

Concomitant HIV, HBV, and TB is poorly studied in sub-Saharan Africa; however a prevalence of 7.5% has been observed in China [11]. Our study has therefore revealed a possibly overlooked health problem that should be studied more in our settings. People with cHIV/HBV/TB may require unique patient care due to the considerable burden on the liver from ATT, HBV, and ART. Our estimated prevalence of cHIV/HBV in Botswana is 28%, including both chronic infections and OBI, which is comparable to prevalence rates that have been reported in Botswana in similar populations [6,9,26]. The prevalence of cHIV/HBV in other sub-Saharan African countries was shown to range from 0% to 27%, with this large range being affected by the management of HBV in different countries [27]. A cHIV/TB prevalence of 10% is reported here, slightly lower than what was previously reported in a previous analysis of the Bomolemo cohort as we did not exclude patients with HBV [20]. Evidence of cHIV/HBV/TB in our participants can therefore be used to propose guidelines for screening PLWHIV for HBV prior to ART and ATT initiation. The five participants who were HBsAg positive but HBcAb negative are suspected to be cases of recent infections; however, this should be confirmed with immunoglobulin M (IgM) testing. Several other reasons, such as the presence of diagnostic escape mutants for the core gene and immunotolerance of core antigen have been discussed to explain these serology results [28].

Baseline participant demographics revealed that there were more females enrolled in the study, which could explain the difference in proportions in gender for PLWHIV and participants with cHIV/HBV. However, this trend was not observed for participants with cHIV/TB, which is also a global trend. In Botswana, incident TB is more prevalent in males than in females [20]. The difference in TB susceptibility between the genders has been discussed to be possibly due to behavioral, physiological, and genetic differences as reviewed by Nhamoyebonde and Leslie [29]. This gender bias is attributed to risk factors such as smoking [30] and protective female sex hormones that modify immune responses and regulate immune cell functions for a controlled mycobacterial infection [31]. Participants with cHIV/HBV/TB exhibited high HIV viral load and low hemoglobin levels. Co-infections are well known to result in worsened prognosis than mono-infections, which is no exception is this study. Concomitant HIV, HBV, and TB can bring in more complications in this case possibly resulting in late viral suppression and a high viral set point. In a previous analysis of the Bomolemo cohort, there was no association between HIV viral load and the risk of TB [20]. Therefore, the association between HIV viral load and multi-infection could be due to the high burden of HBV infection in this cohort. A larger South African study, however, showed HIV viral load as a risk factor for TB [32]. The present study observed the lowest median hemoglobin levels among participants with cHIV/HBV/TB possibly due to TB. Low hemoglobin level has been shown to be a predictor of incident TB in Bomolemo participants [20] and in South African patients during long-term ART [33]. The association of HBV and low hemoglobin levels should be explored further. Participants with cHIV/TB had the lowest BMI, which could be attributed to nutritional deficiency due to TB infection. Nutritional deficiency, resulting in low BMI, was found to be high in pulmonary TB patients [34], while an overweight BMI is shown to significantly reduce risk for TB [35].

With regards to liver enzyme levels, the majority of our study participants had normal liver enzyme levels at baseline. A high incidence of abnormal liver function tests (LFTs) was observed among participants with cHIV/HBV/TB in China [11]. In contrast, in our study, ALT and AST data were only available at baseline, and there was no statistically significant difference in the median liver enzyme levels between all groups. However, there is a possibility of advancing to more toxic grades, which could have been assessed with longitudinal follow-up of liver enzyme tests as more individuals initiate ART. Fibrosis grading in our study showed that participants with cHIV/HBV/TB had a higher proportion of patients with APRI score of greater or equal to 0.5, a low cutoff point for significant liver fibrosis. At this cutoff point, the sensitivity and specificity for the detection of fibrosis is 78% and 68%, respectively. At a cutoff of 1.5, the sensitivity and specificity for the detection of fibrosis is 36% and 92%, respectively [25]. These non-invasive tests are utilized in most resource-limited settings but may miss some cases. Local ALT and AST reference ranges are yet to be defined specifically for men and women in our setting. A recent study, using African reference ranges versus American reference ranges emphasized the need to have specific ranges for different populations to avoid overestimation or underestimation of abnormalities [36]. FibroTest and FibroScan may be preferred over APRI and FIB-4 but may not be suitable for resource-limited settings. The upper cutoff was not pursued due to the modest sample size. Other studies have revealed an association between concomitant HIV/HBV and liver fibrosis [37,38], while one has shown TB to be a risk factor for liver fibrosis [39]. In patients suspected of having cirrhosis (APRI > 1), all had high HIV viral loads and those with HBV had detectable HBV viral loads at baseline, suggesting active HBV replication resulting in liver damage. The association between HBV and HIV viral load with liver fibrosis has been described previously [40]. 

We also assessed immune non-response in these patients, defined as a less than 20% gain in CD4+ T-cell count at 6 months of follow-up with viral suppression at less than 400 copies/mL. The lack of immune non-responders in the HIV/TB cohort could be due to the general small sample size as well as our definition of non-responders. There is currently no consensus on the definition of INRs, defined as reviewed by Yang et al. [41]. In light of this, cHIV/HBV was previously shown to result in slow CD4+ T-cell recovery in these patients compared to PLWHIV only [9,42]. TB also results in slow CD4+ T-cell recovery even after ART initiation [43,44,45]. In one South Africa cohort of 15,646 adults, there was no difference in immune restoration between participants with cHIV/TB and those without TB after ART initiation [46]. The differences in observations from these studies could be attributed to factors such as sample size, immune reconstitution definition, and cofounding factors among others.

Our study bears the limitation of a lack of longitudinal liver enzyme tests. This limits our analysis in terms of assessing for incidence of liver damage in each group of participants, particularly with initiation of ART. A larger population size would have also allowed for other fibrosis cutoff points in the analysis.

## 5. Conclusions

To our knowledge, this is the first study in Botswana to report the prevalence of cHIV/HBV/TB prior to ART initiation. Participants with cHIV/HBV/TB had low hemoglobin levels and high HIV viral loads. Patients with undiagnosed HBV who initiate ATT before ART may have poorer viral hepatitis health outcomes such as developing chronicity, advancing to liver fibrosis, cirrhosis, and possibly hepatocellular carcinoma without any intervention. The study has revealed the need to screen for HBV in people with cHIV/TB infection, as this will lead to better patient management. 

## Figures and Tables

**Table 1:** Participant baseline demographics and clinical characteristics.

Variable	HIV *N* = 170	HIV/HBV *N* = 84	HIV/TB *N* = 30	HIV/HBV/TB *N* = 16	*p*-Value
Age years, median (IQR)	37 (31–43)	35 (31–41)	37 (33–45)	39 (34–45)	0.430
Gender, *n* (%)					
Female	117 (68.8)	54 (64.3)	13 (43.3)	8 (50.0)	
Male	53 (31.2)	30 (35.7)	17 (56.7)	8 (50.0)	0.034 *
^#^HBcAb, *n* (%)					
Negative	90 (53.5)	25 (30.9)	16 (55.2)	3(18.7)	
Positive	79 (46.7)	56 (69.1)	13 (44.8)	13(81.3)	0.001 *
^#^ Hemoglobin g/dL median (IQR)	11.5 (10.2–13)	11.6 (9.9–12.8)	12.1 (9.8–13.5)	9.4 (8.0–11.4)	0.025 *
^#^ BMI, median kg/m^2^ (IQR)	22.2 (19.2–25.1)	21.2 (19.3–25.4)	19.1 (17.6–21.2)	22.9 (18.5–23.6)	0.011 *
^#^ CD4 cells/mm^3^, median (IQR)	168 (88–236)	174 (93–240)	129 (82–230)	91 (31–182)	0.101
^#^ HIV viral load, cps/mL, median (IQR)	5.1 (4.6–5.5)	5.0 (4.5–5.5)	5.3 (4.7–5.7)	5.8 (5.4–5.9)	0.004 *
^#^ ALT, U/L, median (IQR)	22 (15–29)	20 (15–30)	18 (15–23)	21 (18–30)	0.562
^#^ AST, U/L, median (IQR)	28 (23–35)	30 (23–41)	30 (26–38)	33 (27–46)	0.161
^#^ FIB-4, median (IQR)	0.97 (0.71–1.30)	1.02 (0.83–1.40)	1.11 (0.85–1.33)	1.37 (0.85–2.22)	0.071
^#^ APRI, median (IQR)	0.26 (0.19–0.36)	0.28 (0.20–0.38)	0.28 (0.23–0.35)	0.36 (0.19–0.58)	0.30

# Analysis for participants with available data, * statistically significant (*p*-values < 0.05 are statistical different for by Kruskal–Wallis test for continuous variables and by Pearson’s chi-squared test for categorical data). HIV: human immunodeficiency virus; HBV: hepatitis B virus; BMI: body mass index; AST: aspartate aminotransferase; ALT: alanine aminotransferase; APRI: AST platelet ratio index; FIB-4: fibrosis-4 index; IQR: interquartile range.

**Table 2:** APRI and FIB-4 scores among participants.

Variable	HIV *N* = 166	HIV/HBV *N* = 82	HIV/TB *N* = 28	HIV/HBV/TB *N* = 16	*p*-Value
APRI, *n* (%)					
≥0.5	16 (9.6)	13 (15.9)	3 (10.7)	6 (37.5)	
<0.5	150 (90.4)	69 (84.1)	25 (89.3)	10 (62.5)	0.013 *
FIB-4, *n* (%)					
≥1.45	34 (20.5)	20 (24.4)	6 (21.4)	7 (43.8)	
<1.45	132 (79.5)	62 (75.6)	22 (78.6)	9 (56.2)	0.202

* Statistically significant (*p*-values < 0.05 are statistical different by Pearson’s chi-squared test for categorical data). HIV: human immunodeficiency virus; HBV: hepatitis B virus; TB: tuberculosis; APRI: AST platelet ratio index; FIB-4: fibrosis-4 index.

**Table 3:** Baseline demographics of participants with APRI score >1 (lower cutoff for cirrhosis).

Patient code	Gender	Age (years)	Infection	HIV Viral Load (copies/mL)	HBV Viral Load (IU/L)	CD4+ T-Cell Count	Hemoglobin g/dL	BMI	APRI
BB1	Female	37	HIV	152,000	N/A	132	7	19	1.02
BB2	Female	34	HIV	8080	N/A	389	13	27	1.03
BB3	Female	23	HIV	>750,000	N/A	254	14	19	1.11
BB4	Female	38	HIV	545,000	N/A	39	9	18	1.51
^#^ BB5	Male	41	HIV/OBI	>750,000	<20	258	14	19	1.22
BB6	Female	50	HIV/OBI	>750,000	<20	-	12	27	8.47
BB7	Male	32	HIV/HBsAg	27,400	3750	91	17	19	2.53
BB8	Female	22	HIV/HBsAg *	31,300	>170,000,000	204	12	22	4.18
BB9	Male	49	HIV/OBI/TB	>750,000	<20	115	12	18	2.98
BB10	Female	45	HIV/OBI/TB	314,000	3930	71	8	31	3.40

OBI: occult hepatitis B virus infection; HBsAg: hepatitis B virus surface antigen positive; BMI: body mass index. * Lost HBsAg; ^#^ Deceased; - data not known; N/A: not applicable; HIV: human immunodeficiency virus; HBV: hepatitis B virus; APRI: AST platelet ratio index; FIB-4: fibrosis-4 index.
